# National Profile and Treatment Outcomes of Patients with Extrapulmonary Tuberculosis in Bénin

**DOI:** 10.1371/journal.pone.0095603

**Published:** 2014-04-22

**Authors:** Serge Ade, Anthony D. Harries, Arnaud Trébucq, Gabriel Ade, Gildas Agodokpessi, Christine Adjonou, Sophie Azon, Sévérin Anagonou

**Affiliations:** 1 National TB Programme, Cotonou, Benin; 2 International Union against Tuberculosis and Lung Disease, Paris, France; 3 London School of Hygiene and Tropical Medicine, London, United Kingdom; The Chinese University of Hong Kong, Hong Kong

## Abstract

**Background:**

In sub-Saharan Africa, there is a dearth of published literature on extrapulmonary tuberculosis (EPTB).

**Objective:**

To describe demographic, diagnostic and HIV-status characteristics of patients with EPTB in Bénin, their treatment outcomes, and among those who completed their treatment in the Centre National Hospitalier de Pneumo-Phtisiologie (CNHP-P), the proportion whose bodyweight increased during treatment.

**Material and Findings:**

This was a retrospective cohort study with comparisons made between EPTB and new smear-positive pulmonary tuberculosis (NPTB) patients diagnosed in the country from January to December 2011. There were 383 EPTB patients (9% of all TB cases) with a mean age of 35 years, male/female ratio of 1.3 and important regional variation. There were significantly more females (p = 0.001), children <15years (p<0.001) and HIV-positive patients (p = 0.005) with EPTB compared with NPTB. Pleural effusion, spinal and lymph node tuberculosis accounted for 66% of all EPTB. Children <15 years represented 16% of cases, with lymph node disease being most common among them (p<0.001). Of 130 EPTB patients registered in CNHP-P, 7% had a confirmed bacteriological/histological diagnosis. There were 331 (86%) patients who successfully completed treatment. More patients with EPTB were lost-to-follow-up compared with NPTB (p<0.001) with all these patients from one region. The best treatment completion rates were in children <15 years (OR:3.5, 95%CI:1.0–14.8) while patients with pleural effusion and ascites had the worst outcomes. Of 72 HIV-coinfected patients, 88% were on antiretroviral therapy (ART). HIV-positive status was associated with poor outcomes while those on ART fared better. In the CNHP-P, more than 80% who completed their treatment showed an increase in bodyweight and this was more evident in HIV-positive compared with HIV-negative patients (p = 0.03).

**Conclusion:**

Patients with EPTB generally do well in Bénin, although the TB Programme would benefit through more attention to accurate diagnosis and earlier start of ART in HIV-infected patients.

## Introduction

Patients with extrapulmonary tuberculosis (EPTB), i.e., tuberculosis (TB) without associated lung involvement, usually receive less priority in national TB Programmes. However, in many countries, their numbers remain either stable or are increasing while numbers of patients with pulmonary disease are decreasing [1]. In populations with a low prevalence of HIV infection, these patients usually represent 15% to 20% of all TB cases. However, this proportion is thought to be higher in populations with a high prevalence of HIV infection [2], the latter mostly occurring in sub-Saharan Africa.

Confirmation of the diagnosis of EPTB, as recommended by the World Health Organization (WHO) [3], is not easy. Diagnosis in many low income countries is often based on presumptive and circumstantial evidence, with the consequence of a possible misdiagnosis [4].

In Bénin, information is limited about this type of TB, especially with regard to epidemiological characteristics and the most predominant forms of EPTB. Furthermore, the treatment outcomes of patients with EPTB are analyzed in the routine programme together with those of patients with smear-negative pulmonary tuberculosis, the latter in a recent study showing higher rates of unsuccessful treatment compared with new smear-positive pulmonary tuberculosis (NPTB) [5]. These treatment outcomes are usually reported quarterly, using standard classifications; and a “treatment completion” is considered a successful outcome [3]. However, treatment can be completed without improvement of the patient’s health condition, particularly if the diagnosis of EPTB is wrong. The disappearance of initial symptoms along with weight gain in patients who have completed treatment might be a better gauge of successful treatment.

In this study, we aimed to describe the pattern of disease in patients registered as EPTB, their treatment outcomes and among those who completed their treatment in the Centre National Hospitalier de Pneumo-Phtisiologie (CNHP-P), the proportion whose weight increased during the course of treatment. Specific objectives were to determine in Bénin for 2011: i) the number (and proportion) of patients recorded as EPTB and NPTB among all TB cases, ii) demographic, clinical characteristics and HIV-status of patients with EPTB and NPTB, iii) treatment outcomes of patients with EPTB and NPTB and the influence of HIV-infection and antiretroviral therapy and iv) the proportion who increased in weight among those who completed their treatment in the CNHP-P.

## Materials and Methods

### Ethics Statements

This study was approved by “Ethics Advisory Group” of the International Union against Tuberculosis and Lung Disease, Paris, France and “Bénin TB Control Programme Coordination”. Because of its retrospective nature, the local Ethics Committee approval (“Comité National d’Ethique pour la Recherche en Santé”) was not required according to the country’s recommendations.

This study uses already collected data, and written informed consent -given by participants was not possible to obtain. Patient records/Information was collected anonymously. Each participant of this study was attributed a unique identifier number, in order to compare data files that have been double entered. After checking and cleaning the database, the unique identifier numbers have been removed and participants were de-identified prior to analysis. There was no way to recognize them. All databases were kept confidential and have been protected with a password, with only access by authorized persons.

### Study Design

This was a retrospective cohort study of all patients recorded as EPTB and NPTB between 1^st^ January and 31^st^ December 2011 in Bénin.

### Setting – General and Study Site, Including TB Programme

Bénin is a West-African country with a population of 9 million and a HIV prevalence rate of 1.1% [6]. Seven to 10% of the 4000 TB patients registered each year have EPTB. There is a national TB Programme which follows the DOTS strategy and uses recognised international criteria for the diagnosis and treatment of TB patients [7]. Diagnosis, registration and care are decentralised to 57 Basic Management Units in the country. Of them, the largest is the CNHP-P of Cotonou, the economic capital. It houses the *Mycobacteria* Reference Laboratory where culture and molecular diagnostics can be performed for the whole the country.

#### Diagnosis and management of NPTB and EPTB in the Bénin TB control Programme

Pulmonary TB diagnosis is made with respect of WHO recommandations [2]. A person who coughs more than 3 weeks is regarded as having presumptive pulmonary tuberculosis. He/she is requested to provide two sputum samples for acid-fast bacilli microscopy. Sputum samples are examined using either auramine-phenol staining and fluorescence microscopy or Ziehl-Neelsen staining with light microscopy. Patients with at least one sputum smear positive for acid-fast bacilli and who have never been previously treated for TB (or less than one month) are registered as NPTB.

A patient is considered as having presumptive EPTB if he/she has symptoms suggestive of TB related to an extrapulmonary site, with a decision then made by a medical doctor to treat with a full course of anti-tuberculosis treatment. Usually, because of difficulties in accessing and obtaining specimens from different biological sites, TB treatment is started based on symptoms and other circumstantial evidence that includes laboratory investigations and radiographic abnormalities. Whenever possible, specimens collected should be submitted for bacteriological investigation (direct microscopy examination or standard culture, or molecular tests). Rarely, these specimens are also analysed in histology laboratories. In terms of registration, for patients who have TB in both pulmonary and extrapulmonary sites, they are classified as having “pulmonary TB”. For patients with TB in several extrapulmonary sites (e.g., lymph nodes and pleural effusion), they are registered according to the most severe form of disease.

All NPTB and EPTB cases are treated with the same standardized first-line anti-tuberculosis regimen. Patients receive daily rifampicin, pyrazinamide, isoniazid and ethambutol for 2 months (initial phase) followed by daily rifampicin and isoniazid for 4 months (continuation phase). Contrary to the treatment of NPTB, EPTB treatment is not directly observed during the intensive phase and anti-tuberculosis drugs are given every two weeks for self administration. Treatment is free and is only provided by the Programme for all TB patients. TB drugs are not available in private pharmacies in the city or country. While sputum microscopy examination is performed during follow-up of NPTB patients, those with EPTB are monitored clinically and in particular body weight is measured and recorded during treatment on personal treatment cards (at two, five and six months).

Standardized treatment outcomes are monitored through the use of treatment cards and registers, and number of cases and outcomes are quarterly reported.

#### TB/HIV co-infection management

Every patient who is diagnosed with TB (including EPTB) is systematically offered an HIV test using rapid tests and following national guidelines [8]. Those who are found to be HIV positive receive in addition cotrimoxazole. Since 2011 and according to recent WHO guidelines, all TB cases (including EPTB) are eligible for antiretroviral therapy (ART) [9]. The first line ART regimen is efavirenz and two other drugs, zidovudine and lamivudine. If there is anemia (Haemoglobin<7 g/dl) or a low platelet count (<75000/l) zidovudine is replaced by stavudine. Cotrimoxazole and ART are provided free of charge.

### Study Patients

All patients recorded as EPTB and NPTB between 1^st^ January and 31^st^ December 2011 in Bénin were included in the study.

### Data Variables, Sources of Data and Data Collection

Variables for NPTB and EPTB patients included: sex, age, residence by region, HIV-status (positive, negative, unknown), cotrimoxazole and ART administration, treatment outcomes (completion, death, loss-to-follow-up, not evaluated). Variables for EPTB patients included in addition:- sites of disease (pleural effusion, spinal, lymph node, bone or joint, ascites, other and not recorded) and weight change during treatment for patients managed in the CNHP-P. Individual data for EPTB patients were collected from TB registers, laboratory TB registers, treatment cards and TB medical personal files into a standardized paper-based study questionnaire. For NPTB patients, data were collected from quarterly and annual reports.

### Analysis and Statistics

Data were double entered into an electronic file (EpiData3.1, EpiData Association, Odense, Denmark). Data were analyzed by frequencies and percentages. Comparisons were made between groups using the chi-square test, odds ratios (OR) and 95% confidence intervals (95% CI) as appropriate. Levels of significance were set at 5%.

## Results

### Numbers, Epidemiological and Diagnostic Characteristics of TB Patients

The number and proportion of patients with EPTB and NPTB diagnosed in the same time period are shown in **Table 1**. Overall, EPTB patients represented 9% of all TB cases (mean age: 34.5 years, male/female ratio: 1.3), with a large variation between regions from 23% in Borgou (North of the country) to 5% in Ouémé (South of the country). All patients were new cases. Demographic characteristics and HIV-status of patients with EPTB and NPTB are shown in **Table 2**. There were significantly more females (p = 0.001), children under 15 years (p<0.001) and HIV-positive patients (p = 0.005) diagnosed with EPTB compared with NPTB.

**Table 1:** Patients with extrapulmonary tuberculosis and new smear-positive pulmonary tuberculosis registered in Bénin and the different regions of the country in 2011.

Place		All TB**	EPTB (%*)	NPTB (%)
Bénin		4320	383 (8.9)	3331 (77.1)
Regions	Atacora	251	18 (7.2)	189 (75.3)
	Borgou	367	58 (23.1)	255 (69.5)
	Zou	567	70 (12.3)	442 (78)
	Atlantique	1646	146 (8.9)	1226 (74.5)
	Mono	664	51 (7.7)	534 (80.4)
	Ouémé	825	40 (4.8)	685 (83)

Note: EPTB: Extrapulmonary tuberculosis; NPTB: New smear-positive pulmonary tuberculosis; TB: Tuberculosis.

* The percentage is calculated from the total number of TB cases diagnosed in each region.

** All TB cases include all patients with new and retreatment TB, irrespective of the site of tuberculosis.

**Table 2:** Demographic characteristics and HIV-status in patients with extrapulmonary tuberculosis and new smear-positive pulmonary tuberculosis, Bénin, 2011.

Characteristics		EPTB (%)	NPTB (%)	p(value)
Sex	Male	216 (56.4)	2158 (64.8)	0.001
	Female	167 (43.6)	1173 (35.2)	0.001
Age (years)	00–14	62 (16.2)	62 (1.9)	<0.001
	15–24	62 (16.2)	608 (18.3)	NS
	25–34	75 (19.6)	1035 (31.1)	<0.001
	35–44	64 (16.7)	743 (22.3)	0.01
	45–54	58 (15.1)	472 (14.2)	NS
	55–64	36 (9.4)	252 (7.6)	NS
	≥65	26 (6.8)	159 (4.8)	NS
HIV-status	Positive	72 (18.8)	449 (13.5)	0.005
	Negative or unknown	312 (81.5)	2882 (86.5)	0.005
Total		383	3331	

Note: EPTB: Extrapulmonary tuberculosis, NPTB: New smear positive pulmonary tuberculosis; TB: Tuberculosis.

The different sites of EPTB are presented in **Table 3**. Pleural effusion, spinal disease and lymph node disease accounted for 66% of all EPTB cases. Demographic characteristics and HIV-status in relation to the different sites of EPTB are shown in **Table 4**. There was no association between gender and different types of EPTB. Children (under 15 years) represented 16% (62/383) of all EPTB cases diagnosed. Lymph node TB was more common in children than adults (p<0.001) while TB pleural effusion was more common in adults (p<0.001). Otherwise, there were no significant associations between age and other sites of EPTB. Compared with other regions, there was significantly more lymph node TB (OR: 2.6, 95%CI: 1.4–4.7, p = 0.001) and bone or joint TB (OR: 4.9, 95%CI: 2.1–11.6, p<0.001) in Zou while there was significantly more TB pleural effusion in the Atlantique region. TB pleural effusion was significantly more common in HIV-positive patients compared with HIV-negative patients (p<0.001).

**Table 3:** Different sites of extrapulmonary tuberculosis in Bénin, 2011.

Sites	n (%)
Pleural effusion	94 (24.5)
Spine	82 (21.4)
Lymph node	77 (20.1)
Bone and Joint	29 (7.6)
Ascites	15 (3.9)
Other *	17 (4.4)
Not recorded	69 (18)
Total	383

Note: * Others include pericardial TB (n = 10), cerebral TB (n = 1), meningeal TB (n = 1), female genital TB (n = 1), male genital TB (n = 3), skin TB (n = 1).

TB: Tuberculosis.

**Table 4:** Epidemiological characteristics and HIV-status in relation to the different sites of extrapulmonary tuberculosis, Bénin, 2011.

Characteristics		Total	Pleural effusion	Spine	Lymph node	Bone/Joint	Ascites	Other *	Not recorded
Sex	Male	216	58 (26.9)	39 (18.1)	42 (19.4)	16 (7.4)	8 (3.7)	12 (5.6)	41 (19)
	Female	167	36 (21.6)	43 (25.7)	35 (21)	13 (7.8)	7 (4.2)	5 (3)	28 (16.8)
Age (Years)	<15	62	3 (4.8)	12 (19.4)	24 (38.7)	8 (12.9)	2 (3.2)	1 (1.6)	12 (19.4)
	≥15	321	91 (28.3)	70 (21.8)	53 (16.5)	21 (6.5)	13 (4)	16 (5)	57 (17.8)
Region	Atacora	18	1 (5.6)	3 (16.7)	5 (27.8)	0 (0)	1 (5.6)	1 (5.6)	7 (38.9)
	Borgou	58	10 (17.2)	19 (32.8)	10 (17.2)	8 (13.8)	2 (3.4)	2 (3.4)	7 (12.1)
	Zou	70	8 (11.4)	18 (25.7)	24 (34.3)	14 (20)	1 (1.4)	0 (0)	5 (7.1)
	Mono	51	3 (5.9)	9 (17.6)	3 (5.9)	3 (5.9)	2 (3.9)	1 (2)	30 (58.8)
	Atlantique	146	69 (47.3)	27 (18.5)	23 (15.8)	1 (0.7)	8 (5.5)	12 (8.2)	6 (4.1)
	Ouémé	40	3 (7.5)	6 (15)	12 (30)	3 (7.5)	1 (2.5)	1 (2.5)	14 (35)
HIV-Status	Positive	72	29 (40.3)	4 (5.6)	15 (20.8)	0 (0)	5 (6.9)	3 (4.2)	16 (22.2)
	Negative	306	65 (21.2)	74 (24.2)	61 (19.9)	29 (100)	10 (3.3)	14 (4.6)	53 (17.3)
	Unknown	5	0 (0)	4 (80)	1 (20)	0 (0)	0 (0)	0 (0)	0 (0)
Total		383	94	82	77	29	15	17	69

Note: * Others include pericardial TB (n = 10), cerebral TB (n = 1), meningeal TB (n = 1), female genital track TB (n = 1), male genital track TB (n = 3), skin TB (n = 1).

TB: Tuberculosis.

The only health facility where confirmation of diagnostic has been assessed was CNHP-P. Of 130 EPTB patients registered and followed-up in this centre, the diagnosis was confirmed for 9 cases (7%): 2 with positive *Mycobacterium tuberculosis* culture and direct smear examination, 1 with positive culture alone, 1 with positive direct smear examination alone and 5 with histology showing tuberculous granuloma. For aspirates of pleural or peritoneal fluid, measurements of protein concentration and cytological analysis were performed for all cases.

### Treatment Outcomes

Treatment outcomes of patients with EPTB and NPTB are presented in **Table 5**. Overall, 86% of patients successfully completed treatment. There were significantly more patients with EPTB who were lost-to-follow-up compared with NPTB (p<0.001). Treatment outcomes in relation to demographic characteristics, sites of disease, HIV-status and ART administration to co-infected patients are shown in **Table 6**. No association was found between gender and treatment outcomes. However, treatment success was higher in children aged <15 years (OR: 3.5, 95%CI: 1.0–14.8, p: 0.02). All patients who were lost-to-follow-up during treatment were from the Atlantique region and patients with pleural effusion or ascites had the worst completion rates.

**Table 5:** Treatment outcomes of patients with extrapulmonary tuberculosis and new smear-positive pulmonary tuberculosis in Bénin, 2011.

Treatment outcomes	EPTB (%)	NPTB (%)	p (value)
Successful completion	331 (86.4)	2988 (89.9) **	0.03
Failure	0 (0)	99 (3)	<001
Death	32 (8.4)	196 (5.9)	NS
Loss-to-follow up	15 (3.9)	27 (0.9)	<0.001
Not evaluated	5 (1.3)	14 (0.4)	0.04
Total evaluated	383	3324 *	

Note: EPTB: Extrapulmonary tuberculosis; NPTB: New smear positive pulmonary tuberculosis.

* 7 patients of the 3331 were wrongly registered as new smear-positive TB and were removed from the cohort analysis one year later.

** 2800 of the 2988 patients were cured with negative sputum smear examination for acid-fast bacilli.

**Table 6:** Treatment outcomes of patients with extrapulmonary tuberculosis in relation to demographic characteristics, regions, HIV status and ART administration in those with HIV coinfection, Bénin, 2011.

Characteristics		Total	Completion	Death	Loss-to-follow-up	Not evaluated
Sex	Male	216	183 (84.7)	17 (7.9)	11 (5.1)	5 (2.3)
	Female	167	148 (88.6)	15 (9)	4 (2.4)	0 (0)
Age (years)	<15	62	59 (95.2)	2 (3.2)	1 (1.6)	0 (0)
	≥15	321	272 (84.7)	30 (9.3)	14 (4.4)	5 (1.6)
Region	Atacora	18	17 (94.4)	1 (5.6)	0 (0)	0
	Borgou	58	47 (81)	10 (17.2)	0 (0)	1 (1.7)
	Zou	70	67 (95.7)	2 (2.9)	0 (0)	1 (1.4)
	Mono	51	47 (92.2)	4 (7.8)	0 (0)	0 (0)
	Atlantique	146	114 (78.1)	14 (9.6)	15 (10.3)	3 (2.1)
	Ouémé	40	39 (97.5)	1 (2.5)	0 (0)	0 (0)
Site	Pleurisy	94	73 (77.7)	11 (11.7)	7 (7.4)	3 (3.2)
	Spine	82	75 (91.5)	4 (4.9)	1 (1.2)	2 (2.4)
	Lymph node	77	70 (90.9)	4 (5.2)	3 (3.9)	0 (0)
	Bone and Joints	29	27 (93.1)	2 (6.9)	0 (0)	0 (0)
	Ascites	15	10 (66.7)	4 (26.7)	1 (6.7)	0 (0)
	Other*	17	14 (82.4)	1 (5.9)	2 (11.8)	0 (0)
	Not recorded	69	62 (89.9)	6 (8.7)	1 (1.4)	0 (0)
HIV-Status	Negative	306	270 (88.2)	21 (6.9)	11 (3.6)	4 (1.3)
	HIV positive on ART	63	52 (82.5)	9 (14.3)	1 (1.6)	1 (1.6)
	HIV positive not on ART	9	4 (44.4)	2 (22.2)	3 (33.3)	0 (0)
	Unknown	5	5 (100)	0 (0)	0 (0)	0 (0)
Total		383	331	32	15	5

Note: * Others include pericardial TB (n = 10), cerebral TB (n = 1), meningeal TB (n = 1), female genital track TB (n = 1), male genital track TB (n = 3), skin TB (n = 1).

TB: Tuberculosis; ART: Antiretroviral therapy.

Finally, of 72 HIV-TB co-infected patients, 99% received cotrimoxazole and 88% were given ART. HIV-positive patients were at higher risk of death than HIV-negative patients during treatment (OR: 2.5, 95%CI: 1.0–5.7, p: 0.02). In HIV-positive patients, those on ART had much better outcomes than those not on ART (82.5% versus 44.4%, p = 0.03). The concomitant administration of ART was also associated with a lower loss-to-follow-up rate (OR: 31, 95%CI: 2.2–922, p: 0.005). There were no other significant differences.

### Weight Variation during Treatment

The change in body weight among patients who completed their treatment in relation to HIV-status in the CNHP-P, the largest basic management unit in Bénin, is shown in **Table 7**. Up to 80% of patients who successfully completed treatment showed an increase in body weight, and this was more apparent among HIV-positive than HIV-negative patients (p = 0.03).

**Table 7:** Treatment completion and change in weight in HIV-positive and HIV-negative patients with extrapulmonary tuberculosis treated in the Centre National Hospitalier de Pneumo-Phtisiologie, Bénin.

Characteristics	All patients	HIV-negative	HIV-positive	p (value)
Extrapulmonary tuberculosis	129	95	34	
Treatment Completion n (%*)	101 (78.3)	74 (77.9)	26 (76.5)	0.86
Increase in Weight n (%**)	82 (81.2)	60 (81.1)	21 (80.8)	0.83
Mean increase in weight (Kg) [95%CI]	5.8 [4.9–6.6]	5.21 [4.2–6.2]	7.3 [5.7–8.9]	0.03

Note:

* The percentage was calculated from the EPTB cases diagnosed.

** The percentage is calculated from the total number of patients who completed their treatment.

95CI = 95 Confidence Interval.

## Discussion

This is the first study in Bénin to describe the epidemiological and diagnostic characteristics and treatment outcomes of EPTB patients. Patients with EPTB constituted less than 10% of all notifications, with some parts of the country showing higher prevalence than others. EPTB appeared to be more common in females, children under the age of 15 years and in those who were HIV-positive. Predominant sites of disease were pleural, spinal and lymph node, with TB lymph node disease being more common in children and pleural effusion being more common in adults, especially those HIV-positive. Diagnostic confirmation was rare even in the largest basic management unit in the country.

Successful treatment completion was good especially in children, although there was a higher rate of loss-to-follow-up when compared with patients who had NPTB. Interestingly, all patients lost to follow-up were from a specific region of Bénin. Patients with pleural effusion and ascites had the worst treatment completion rates. Patients with HIV-associated TB remained at higher risk of death compared with those who did not have HIV-infection, although those on ART fared much better. Finally, in CNHP-P where weight during the treatment has been collected, there was a significant weight gain amongst those who completed treatment, with the change in weight being more evident in patients who were HIV-positive compared with those who were not HIV-infected.

The strengths of this study were that it involved the whole population and it was therefore nationally representative of the epidemiology of EPTB in Bénin. There was no need for any sampling framework. In addition, basic management units are regularly supervised every three months by the TB control Programme coordination, and during these supervisory visits, quarterly reports and patients’ personal data are systematically reviewed and if necessary, corrections are made. Limitations related to the operational nature of the study which used already collected data from registers, medical files and quarterly reports which are difficult to validate when conducting a retrospective record review. In spite of regular supervision, some errors could also remain in the consulted documents.

There were important variations in the proportion of EPTB patients in the different regions of the country; and this could be related to the implication of medical doctors in the diagnosis of this form of TB. In Borgou region, where the highest proportion of EPTB cases was reported from, the large majority of these patients were diagnosed in two well frequented hospitals because of their good reputation (a confessional centre and a teaching hospital).

The proportion of patients diagnosed with EPTB in Bénin was lower than that reported from other parts of world [1], [10]–[15], and few diagnoses were confirmed. Lack of appropriate and specific diagnosis in EPTB in sub-Saharan Africa remains a continuing source of debate [4]. There are recommendations that the diagnosis of EPTB should be based on at least one specimen with confirmed *Mycobacterium tuberculosis* (using microscopy examination, culture or rapid molecular testing), histological or strong clinical evidence consistent with active EPTB, followed by a clinician decision to treat with a full course of tuberculosis chemotherapy [3].

However, in resource-poor countries like Bénin, following these recommendations is difficult. Obtaining the diagnosis is difficult for several reasons, such as lack of specialised physicians, microbiologists and pathologists, weak laboratory infrastructure and poor access to radiography and other more sophisticated imaging technologies such as ultrasound and computerised tomography scans.

The finding of females, children and HIV-positive patients having a higher prevalence of EPTB is in line with reports from other countries [1], [12], [16], [17]. For children, the predominant site of TB reported in the literature is usually lymph node disease [1], although in adults a wide variety of patterns is reported from other countries which may depend to a large extent on resources available for diagnosis [16], [18]–[25]. In Bénin, we found few patients with TB meningitis, while the proportion diagnosed in others studies has been higher [23], [24]. This may be due to good coverage of BCG immunization in Bénin and also the difficulties in establishing the diagnosis in this type of disease [22], [26], [27].

Although successful treatment completion was reasonable, this could be improved by reducing the losses-to-follow-up, especially in Atlantique region, where Cotonou, the economic capital city of the country, is located. One of the reasons for patients being lost-to-follow-up may be misdiagnosis, and this has been found in other parts of Africa. Patients with other pathology such as lung cancer, mesothelioma or systemic disease may be wrongly treated with anti-tuberculosis drugs without improvement, persuading them to seek alternative opinions and stopping treatment. Another reason is that some patients reported as lost-to-follow-up may have died, and this has also been found in other studies [28]. Finally more attention needs to be paid to accurate diagnosis of patients with pleural effusion and ascites, the latter especially having a wide differential diagnosis that includes chronic liver disease.

HIV-positive patients with EPTB were at higher risk of death than HIV-negative patients, despite good coverage with ART. Often, HIV-infected patients with EPTB present with advanced immune suppression with CD4 counts <200 cells/µl, and hence their classification as a WHO Stage 4 AIDS defining disease. As a result, patients can also be affected by other opportunistic diseases that can result in death [29], [30]. The time of start of ART in relation to start of anti-TB treatment is an important factor that determines mortality, and late initiation of ART is associated with increased case fatality [9]. A weakness of this study was the lack of information about when ART was started.

Finally, we assessed whether patients who completed treatment increased their weight, the latter adding to the evidence to support a successful treatment completion. The large majority of patients who completed their treatment showed an increase in body weight, especially in HIV-positive patients, and this has been found elsewhere. As recommended in guidelines, weight should be regularly measured during the course of treatment [4], [31].

The findings of this study have several implications for the Bénin TB control Programme. First, there is a need to improve the diagnosis of EPTB in the country and to perform appropriate examinations when needed such as mycobacterial culture on tissue and other specimens. The introduction of rapid molecular tests such as Xpert MTB/RIF should be considered especially for the more difficult-to-diagnose cases of EPTB [32], [33]. Second, the follow-up of patients during treatment must improve especially in the Atlantique region where there was high loss-to-follow-up. Third, more attention needs to be paid to getting HIV-infected patients on to ART as soon as possible and to ensure that the time of start of ART in relation to start of anti-TB treatment is well documented.

## Conclusion

The study has shown that the proportion of patients diagnosed and registered with EPTB was low and below 10%. Treatment outcomes of EPTB cases were satisfactory, although the high loss-to-follow-up in the Atlantique region of the country needs to be rectified. The TB control Programme in Bénin would benefit through more attention to accurate diagnosis of EPTB and earlier start of ART in HIV-infected patients.

## References

[1] Sandgren A, Hollo V, van der Werf MJ (2013) Extrapulmonary tuberculosis in the European Union and European Economic Area, 2002 to 2011. Euro Surveill 18(12): pii = 20431. Available: http://www.eurosurveillance.org/ViewArticle.aspx?ArticleId=20431. Accessed 2013 Dec 9. 23557943

[2] Tuberculosis Coalition for Technical Assistance (2009) International Standards for Tuberculosis Care (ISTC), 2^nd^ edition. Tuberculosis Coalition for Technical Assistance, The Hague. Available: http//www.istcweb.org. Accessed 12^Th^ December 2013.

[3] WHO (2009) Treatment of tuberculosis Guidelines 4^Th^ edition. WHO/HTM/TB/2009.420.

[4] Harries AD, Hargreaves NJ, Kwanjana JH, Salaniponi FM (2003) The diagnosis of extra-pulmonary tuberculosis in Malawi. Trop Doct 33: 7–11. 12568510 10.1177/004947550303300106

[5] Ade S, Harries AD, Trébucq A, Hinderaker SG, Ade G, et al (2013) National profile and treatment outcomes of adult smear-negative pulmonary tuberculosis patients in Benin. Trans R Soc Trop Med Hyg 107: 783–788 10.1093/trstmh/trt092 24218414

[6] UNAIDS (2013) Global report: UNAIDS report on the global AIDS epidemic 2013. UNAIDS: Geneva, Switzerland. UNAIDS.

[7] WHO IUATLD (2001) KNCV (2001) Revised international definitions in tuberculosis control. Int J Tuberc Lung Dis 5(3): 213–215. 11326818

[8] National Tuberculosis Control Programme (2009) National tuberculosis guide. Cotonou, Benin: NTP. Available: http://www.pnt-benin.bj/spip.php?article48. Accessed 2013 Dec 9.

[9] WHO (2010) Antiretroviral therapy for HIV infection in adults and adolescents. Recommendations for a public health approach. Geneva, Switzerland. World Health Organization. 23741771

[10] Rock RB, Sutherland WM, Baker C, Williams DN (2006) Extra-pulmonary tuberculosis among Somalis in Minnesota. Emerg Infect Dis 12: 1434–1436. 17073097 10.3201/eid1209.050295 PMC3294725

[11] te Beek LAM, van der Werf MJ, Richter C Borgdorff MW (2006) Extrapulmonary tuberculosis by nationality, the Netherlands, 1993–2001. Emerg Infect Dis 12: 1375–1382. Available: http//www.cdc.gov/eid. Accessed 9^th^ December 2013. 10.3201/eid1209.050553 PMC3294726 17073086

[12] Peto HM, Pratt RH, Harrington TA, LoBue PA, Armstrong LR (2009) Epidemiology of extrapulmonary tuberculosis in the United States, 1993–2006. Clin Infect Dis 49: 1350–1357. 19793000 10.1086/605559

[13] Kruijshaar ME, Abubakar I (2009) Increase in extrapulmonary tuberculosis in England and Wales 1999–2006. Thorax 64: 1090–1095 10.1136/thx.2009.118133 19850965

[14] Garcia-Rodriguez JF, Alvarez-Diaz H, Lorenzo-Garcia MV, Marino-Callejo A, Fernandez-Rial A, et al (2011) Extrapulmonary tuberculosis: epidemiology and risk factors. Enferm Infecc Microbiol Clin 29(7): 502–509. 21570159 10.1016/j.eimc.2011.03.005

[15] Mazza-Stalder J, Nicod L, Janssens JP (2012) Extrapulmonary tuberculosis. Rev Mal Respir 29(4): 566–578 10.1016/j.rmr.2011.05.021 22542414

[16] Lin JN, Lai CH, Chen YH, Lee SSJ, Tsai SS, et al (2009) Risk factors for extra-pulmonary tuberculosis compared to pulmonary tuberculosis. Int J Tuberc Lung Dis 13(5): 620–625. 19383196

[17] Yang Z, Kong Y, Wilson F, Foxman B, Fowler AH, et al (2004) Identification of Risk Factors for extrapulmonary tuberculosis. Clin Infect Dis 38: 199–205. 14699451 10.1086/380644

[18] Leeds IL, Magee MJ, Kurbatova EV, del Rio C, Blumberg HM, et al (2012) Site of extrapulmonary tuberculosis is associated with HIV infection. Clin Infect Dis 55(1): 75–81. 22423123 10.1093/cid/cis303 PMC3493181

[19] Gunal S, Yang Z, Agarwal M, Koroglu M, Arici ZK, et al. (2011) Demographic and microbial characteristics of extrapulmonary tuberculosis cases diagnosed in Malatya, Turkey, 2001–2007. BMC Public Health 11: 154. Available: http://www.biomedcentral.com/1471-2458/11/154. Accessed 2013 Dec 9. 10.1186/1471-2458-11-154 PMC3060117 21385458

[20] Gonzalez OY, Adams G, Teeter LD, Bui TT, Musser JM, et al (2003) Extrapulmonary manifestations in a large metropolitan area with a low incidence of tuberculosis. Int J Tuberc Lung Dis 7: 1178–1185. 14677893

[21] Özvaran MK, Baran R, Tor M, Dilek I, Demiryontar D, et al (2007) Extrapulmonary tuberculosis in non-human immunodeficiency virus-infected adults in an endemic region. Ann Thorac Med 2(3): 118–121 10.4103/1817-737.33700 19727358 PMC2732087

[22] Ozbay B, Uzun K (2002) Extrapulmonary tuberculosis in high prevalence of tuberculosis and low prevalence of HIV. Clin Chest Med 23(2): 351–354. 12092030 10.1016/s0272-5231(02)00002-3

[23] Kourbatova EV, Leonard MK Jr, Romero J, Kraft C, del Rio C, et al (2006) Risk factors for mortality among patients with extrapulmonary tuberculosis at an academic inner-city hospital in the US. Eur J Epidemiol 21(9): 715–721. 17072539 10.1007/s10654-006-9060-7

[24] Savina TA, Suprun TIu (2007) The pattern of extrapulmonary tuberculosis according to the materials of Saint Petersburg City Tuberculosis Hospital Two and the problems in delivery of health care. Probl Tuberk Bolezn Legk (7): 12–15. 17718068

[25] Jamtsho T, Harries AD, Malhotra S, Wangchuk D, Dophu U, et al (2013) The burden and treatment outcomes of extra-pulmonary tuberculosis in Bhutan. PHA 3(1): 38–42. 26392994 10.5588/pha.12.0085 PMC4463083

[26] Ministère de la Santé (2012) Annuaire des statistiques sanitaires de l’année 2012. Cotonou, Bénin: Ministère de la Santé. [French] Available: http://www.beninsante.bj/spip.php?article66. Accessed 2013 Dec 12.

[27] Trunz BB, Fine PEM, Dye C (2006) Effect of BCG vaccination on childhood tuberculous meningitis and military tuberculosis worldwide: a meta-analysis and assessment of cost-effectiveness. Lancet 367: 1173–1180. 16616560 10.1016/S0140-6736(06)68507-3

[28] Kruyt ML, Kruyt ND, Boeree MJ, Harries AD, Salaniponi FM, et al (1999) True status of smear-positive pulmonary tuberculosis defaulters in Malawi. Bull World Health Organ 77(5): 386–391. 10361755 PMC2557676

[29] Kwan CK, Ernst JD (2011) HIV and Tuberculosis: a deadly human syndemic. Clin Microbiol Rev 24(2): 351 10.1128/CMR.00042-10 21482729 PMC3122491

[30] Kingkaew N, Sangtong B, Amnuaiphon W, Jongpaibulpatana J, Mankatittham W, et al (2009) HIV-associated extrapulmonary tuberculosis in Thailand: Epidemiology and risk factors for death. Int J Infect Diseases 13: 722–729. 19196530 10.1016/j.ijid.2008.11.013

[31] Bernabe-Ortiz A, Carcamo CP, Sanchez JF, Rios J (2011) Weight variation over time and its association with tuberculosis treatment outcome: A longitudinal analysis. PLoS ONE 6(4): e18474 10.1371/journal.pone.0018474 21494617 PMC3072983

[32] Lawn SD, Zumla AI (2012) Diagnosis of extrapulmonary tuberculosis using the Xpert MTB/RIF Assay. Expert Rev Anti Infect Ther 10(6): 631–635. 22734954 10.1586/eri.12.43 PMC3605769

[33] Vadwai V, Boehme C, Nabeta P, Shetty A, Alland D, et al (2011) Xpert MTB/RIF: a new Pillar in diagnosis of extrapulmonary tuberculosis. J Clin Microbiol 49(7): 2540–2545. 21593262 10.1128/JCM.02319-10 PMC3147857

